# Henri Meige (1866–1940)

**DOI:** 10.1007/s00415-017-8575-z

**Published:** 2017-07-29

**Authors:** Michał K. Owecki, Halina Bogusz, Anita Magowska

**Affiliations:** Department of History of Medical Sciences, Poznań University of Medical Sciences, ul. Przybyszewskiego 37A, Poznań, Poland

Henri Meige was a French neurologist now known for the eponymous “Meige’s syndrome”, which is a neurological condition characterized by blepharospasm and orofacial dystonic movements.

Born on February 11, 1866 in Moulins-sur-Allier in France, into a family with a rich medical tradition, Meige represented the fourth generation of doctors. He graduated from high school in his hometown, moved to Paris to study medicine and, following his graduation, qualified for a position at Salpêtrière Hospital. His medical training was conducted under the guidance of Jean-Martin Charcot, the Head and the Chair of the Clinic of Nervous System Diseases at Salpêtrière. Meige had the advantage of being one of Charcot’s last interns and, until the end of his life, Meige remained under the great influence of his master’s genius. A few years after Charcot’s death, Meige wrote a short monograph, glorifying not only Charcot’s artistic interests, but also the artistry of his clinical skills and unmistakable diagnostic accuracy; the book was re-edited in 1925 [1].

In 1893, the year of Charcot’s death, Meige obtained his doctorate with a thesis entitled “The Study of Certain Neuropathological Travelers: The Wandering Jew at the Salpêtrière”. Meige prepared his doctoral dissertation under Charcot’s tutelage, basing it on his and Charcot’s observations of Jews hospitalized at the Salpêtrière. However, he also incorporated some conclusions derived from non-academic papers on “wandering Jews”; part of which comprised hideously anti-Semitic trends. Meige noticed in his Jewish patients an irrepressible need to wander continuously, “from city to city, from clinic to clinic” in a search for “an efficacious remedy”. However, at the same time, he also noted these patients’ tendency to withdraw swiftly from any proposed therapy with new medications which were allegedly much more effective. Diagnosing “an obsession with constant traveling” or a compulsive unsteadiness in his patients, Meige reached the conclusion that this was a kind of putatively congenital and race-specific disease touching the Jewish population, and that racial factors were responsible for their inability to settle in a certain place or become part of a certain nation. Words that, in the context of the future Holocaust, are abhorrent.

Fortunately, Meige’s doctoral analysis is not what represents his considerable contribution to neurology [2]. In 1894, Meige left Salpêtrière and followed his new mentor, Édouard Brissaud, to continue his neurological work at the Saint-Antoine Hospital in Paris.

Meige’s interest at that time was focused on acromegaly and the skeletal changes caused by the disease. In 1895, with Brissaud’s cooperation, Meige accurately concluded that gigantism in teenagers and acromegaly in adults shared the same pathogenesis and was actually the same disease entity, although with a different age of onset [3].

Later, in collaboration with Eugene Feindel, Meige took up a subject that Georges Gilles de la Tourette had dealt with a decade or so earlier. In 1885, Tourette precisely reported the case of the Marquise de Dampierre, who was touched by a disease presenting with a composition of vocal and motor tics. Her illness began in childhood and the tics increased in number and severity over time. Although Tourette was awarded the honour of having the syndrome named after him, he proposed an explanation of the disease pathogenesis that reflected the spirit of the time: tics were a consequence of inherited nervous system degeneration due to an accumulation of the effects of immoral behavior in previous generation [4, 5].

In 1902, Meige presented his conceptions on tic pathogenesis, symptomatology and treatment in a monumental monograph entitled “Les tics et leur traitement” [6]. The book, which was translated into English in 1907 by a famous British neurologist, Kinnier Wilson, became a fundamental source of knowledge for the next half century and the basis for theoretical assumptions about convulsive vocal and motor tics. Meige considered tics to be a psychological pathology that developed in people who inherited susceptibility. According to Meige, poor will and weak self-control resulted in the conversion of bad habits formed during childhood into persistent convulsive tics. Tics were initially performed to gain a kind of somatic or emotional relief, but with time and repetition became habitual in a psychologically predisposed individual, even if the provoking stimulation ceased. Thus, Meige claimed, a congenital degenerative psychological disorder presenting with “hereditary weakness of the will” played a crucial role in the development of convulsive behavior. An ability to suppress tics temporarily, accompanied by personality changes, served as undeniable proof of their psychogenic origin [6].

Meige continued his studies on involuntary movements and, in an article written in 1910, produced a report on the combination of blepharospasm and facial, mandibular, oral, lingual, and laryngeal spasms. Paradoxically, this short paper, with the first precise description of blepharospasm and orofacial dystonia, became his most important contribution to neurology and “Meige’s syndrome” remains one of the most recognizable medical eponyms in neurological practice [7].

The tragedy of the First World War brought new challenges for the medical staff of the time. Along with others, French neurologists had to face the consequences of a large-scale war never experienced before: millions of soldiers suffered severe psychological and somatic injuries and new types of weapons meant new types of wounds. Throughout the Great War, Meige continued to carry out his research work, combining the care of injured soldiers with his scientific interests. Together with his Salpêtrière colleague Pierre Marie, Meige concentrated on the pathogenesis and clinical spectrum of neuropathy, using a modern method of electrical nerve stimulation. Their studies contributed new insights into knowledge of diseases of the peripheral nervous system. In 1915, Meige and Marie published a paper on localizing symptomatology in neuropathies and nerve injuries [8].

Clinical neurology was not the only love of Meige’s life—he also dedicated himself to art. When bored with long lectures and discussions, Meige used to follow his attraction to art by unobtrusively escaping from medical congresses to visit local galleries and museums. Guided by another of Charcot’s former assistants, Professor Paul Richer, Meige developed his talent and gained a position at the École des Beaux-Arts—a famous art school in Paris. His artistic skills and invention helped him to assume the position of editor of two important French medical journals at the turn of the twentieth century: “Nouvelle iconographie de la Salpêtrière” and “Revue Neurologique”. Moreover, many of his articles were illustrated with drawings of his own creation. He also—surprisingly for a neurologist—had considerable knowledge of entomology and botany, frequently sketching pictures of the insects or plants he saw and described to his friends. In 1922, Meige succeeded Richer as a professor and the Head of the Chair of Artistic Anatomy at the École des Beaux-Arts.

In his friends’ memoirs, Meige is recalled as a tall gentleman, who listened attentively to others, had a long face, was rather distant, calm and courteous, but liked by his colleagues and loved by his students; because of his musketeer mustache, his co-workers facetiously called him the “Count of Fère”, a title given to Athos, one of “The Three Musketeers” in the novel by Alexandre Dumas [9].

Henri Meige passed away on September 29, 1940, but lives on in the vocabulary of neurology in the eponymous syndrome he had described 30 years earlier [10].
